# An Outbreak of Type Π Vaccine-Derived Poliovirus in Sichuan Province, China: Emergence and Circulation in an Under-Immunized Population

**DOI:** 10.1371/journal.pone.0113880

**Published:** 2014-12-11

**Authors:** Hai-Bo Wang, Gang Fang, Wen-Zhou Yu, Fei Du, Chun-Xiang Fan, Qing-Lian Liu, Li-Xin Hao, Yu Liu, Jing-Shan Zheng, Zhi-Ying Qin, Wei Xia, Shi-Yue Zhang, Zun-Dong Yin, Qiong Jing, Yan-Xia Zhang, Rong-Na Huang, Ru-Pei Yang, Wen-Bin Tong, Qi Qi, Xu-Jing Guan, Yu-Lin Jing, Qian-Li Ma, Jin Wang, Xiao-Zhen Ma, Na Chen, Hong-Ru Zheng, Yin-Qiao Li, Chao Ma, Qi-Ru Su, Kathleen H. Reilly, Hui-Ming Luo, Xian-Ping Wu, Ning Wen, Wei-Zhong Yang

**Affiliations:** 1 Chinese Center for Disease Control and Prevention, Nanwei Rd 27#, Xicheng District, Beijing 100050, PR China; 2 Peking University Clinical Research Institute, Xueyuan Rd 38#, Haidian District, Beijing 100191, PR China; 3 Sichuan Provincial Center for Disease Control and Prevention, Zhongxue Road 6#, Chengdu City, Sichuan Province 610041, PR China; 4 Ngawa Tibetan and Qiang Autonomous Prefectural Center for Disease Control and Prevention, Meigu Street 178#, Maerkang County, Ngawa Tibetan and Qiang Autonomous Prefecture, Sichuan Province 624000, PR China; 5 Chengdu City Center for Disease Control and Prevention, Longxiang Rd 4#, Chengdu City, Sichuan Province 610041, PR China; 6 National Center for AIDS/STD Control and Prevention, Chinese Center for Disease Control and Prevention, Nanwei Rd 27#, Xicheng District, Beijing 100050, PR China; The Scripps Research Institute, United States of America

## Abstract

**Background:**

During August 2011–February 2012, an outbreak of type Π circulating vaccine-derived poliovirus (cVDPVs) occurred in Sichuan Province, China.

**Methods:**

A field investigation of the outbreak was conducted to characterize outbreak isolates and to guide emergency response. Sequence analysis of poliovirus capsid protein VP1 was performed to determine the viral propagation, and a coverage survey was carried out for risk assessment.

**Results:**

One clinical compatible polio case and three VDPV cases were determined in Ngawa County, Ngawa Tibetan and Qiang Autonomous Prefecture, Sichuan Province. Case patients were unimmunized children, 0.8–1 years old. Genetic sequencing showed that the isolates diverged from the VP1 region of the type Π Sabin strain by 5–12 nucleotides (nt) and shared the same 5 nt VP1 substitutions, which indicate single lineage of cVDPVs. Of the 7 acute flaccid paralysis cases (all>6 months) reported in Ngawa Prefecture in 2011, 4 (57.1%) cases (including 2 polio cases) did not receive oral attenuated poliovirus vaccine. Supplementary immunization activities (SIAs) were conducted in February–May, 2012, and the strain has not been isolated since.

**Conclusion:**

High coverage of routine immunization should be maintained among children until WPV transmission is globally eradicated. Risk assessments should be conducted regularly to pinpoint high risk areas or subpopulations, with SIAs developed if necessary.

## Background

Since the World Health Assembly launched the Global Polio Eradication Initiative in 1988, global polio eradication activities have reduced wild poliovirus (WPV) cases from an estimated 350,000 in 1988 to 223 reported cases in 2012 [1], and indigenous transmission of type Π WPV had been interrupted globally since 1999 [2]. The number of countries in which WPV transmission has never been interrupted decreased from 125 to 3 during the same period [3]. Despite significant progress toward global eradication of polio, many previously polio-free countries remain at risk for WPV importation from the remaining endemic countries [4]–[6].

Historically, poliomyelitis had been endemic and widespread in China, with approximately 20,000 paralytic cases reported annually [7]. The number of poliomyelitis cases declined dramatically through a combination of routine immunization and supplementary immunization activities (SIAs). The last indigenous case of WPV was reported in September 1994 and in October 2000, China was certified as polio free [7], [8]. China, which shares border with two of three endemic countries, has experienced three instances of WPV importation: 1995 and 1996 in Yunnan Province [9], and 1999 in Qinghai Province [10]. Moreover, after being polio-free for more than 10 years, on August, 2011, an outbreak was confirmed in Xinjiang Uygur Autonomous Region, China following importation of type I WPV originated from neighboring Pakistan [11].

Four doses of oral attenuated poliovirus vaccine (OPV) are recommended at ages 2, 3, and 4 months and 4 years in the schedule of national immunization program. The main advantages of OPV are ease of administration, relatively inexpensive, passively immunizing persons who do not receive vaccine directly, and inducing greater intestinal immunity than that conferred by inactivated poliovirus vaccine. However, on rare occasions, OPV has been known to carry infrequent risks of vaccine-associated paralytic poliomyelitis (VAPP) and vaccine-derived polioviruses (VDPVs). VDPVs are further categorized as circulating VDPVs (cVDPVs) when there are two or more poliomyelitis cases infected by genetically related VDPVs [12]. The risk of cVDPVs emergence appears to be highest for the Sabin type Π OPV strain [12]–[14], particularly in areas with poor sanitation and tropical or subtropical climates [15], however, the key risk factor for cVDPVs emergence and spread is low population immunity [14], [16]. VDVPs may present a potential risk to China's polio-free status. An outbreak of type Ι cVDPVs was reported in Guizhou Province, China in 2004 [7].

We report another outbreak of cVDPVs caused by type Π VDPVs strain in Ngawa County, Ngawa Tibetan and Qiang Autonomous Prefecture, Sichuan Province, during August 2011–February 2012. The present report describes the results of epidemiological and laboratory investigations, and control efforts regarding the cVDPVs outbreak.

## Methods

### Setting and population

Sichuan is a mountainous province, situated in southwest China and bordered by Chongqing, Qinghai, Gansu, Shan′xi, Yunnan, Guizhou and Tibet Provinces. In 2012 the population was estimated to be 80.8 million with 13.4 million children younger than 15 years of age; the average population density is 267 persons/km^2^, and the annual birth rate is approximately 9.9‰. The majority (56%) of the population live in rural villages; 4.9 million (6.1%) are ethnic minorities. Ngawa County lies in the northwest of Sichuan Province and at the juncture of Sichuan, Gansu, and Qinghai provinces. It consists of 19 townships and is located in Ngawa Tibetan and Qiang Autonomous Prefecture, one of 21 prefectures in Sichuan. The majority (90%) of the 50,000 residents are Tibetan minorities.

### Case ascertainment and definition

Case patients with paralytic poliomyelitis were identified through acute flaccid paralysis (AFP) cases surveillance system, which was established in 1993 by the Chinese government in accordance with World Health Organization (WHO) recommendations. Staffs at the county-level Center for Disease Control and prevention (CDC) routinely investigate AFP cases reported by hospitals and clinics, collect stool specimens and assess residual paralysis 60 days after the onset of paralysis. Clinical and epidemiological information on AFP cases are abstracted from medical records and/or obtained from case investigations; a standard case investigation form is used in China. AFP surveillance quality is monitored by WHO performance standards [17].

An AFP case is defined as any child younger than 15 years of age presenting with AFP, or any person at any age with paralytic illness if poliomyelitis is suspected. According to National AFP cases surveillance guidelines, AFP cases are classified as follows: WPV case, a person with AFP for whom a stool specimen tested positive for WPV by virology; clinical compatible polio case, an AFP case who tested negative for WPV on inadequate stool specimens, but was determined to be polio-compatible by the provincial Polio Expert Committee (PEC) of China after the standard 60-day follow-up examination; non-polio AFP case, person with AFP who tested negative for poliovirus on adequate stool specimens or who were judged by provincial PEC to not be polio-compatible. In accordance with WHO current recommendations, a VDPV case was defined as an AFP case from whom Sabin-related poliovirus (type Π with ≥6 nucleotides (nt) VP1 substitutions; type Ι and Ш with ≥10 nt VP1 substitutions) was isolated from ≥1 stool specimen without WPV isolated, and who was determined to be polio compatible by provincial PEC.

### Rapid Assessment of Vaccination Coverage

After confirmation of VDPVs cases, a convenient survey for rapid assessment of OPV coverage was conducted for children younger than 5 years of age in Ngawa County. OPV immunization status was determined by checking hospital immunization records or immunization cards. Related information from caregivers was also collected.

### Epidemiological investigation on close contacts

The contacts of VDPVs cases, including people who lived in the same household, neighbors, hospital contacts and classmates were also investigated. Demographic characteristics, immunization records, contact information and fecal specimens were collected.

### Isolation and characterization of poliovirus isolates

Stool specimens were forwarded to the provincial polio laboratory where viral isolation was performed on L20B and RD cell cultures and viral isolates were identified by micro-neutralization assay. Poliovirus isolates were forwarded to the National Polio Laboratory where intratypic differentiation was performed by polymerase chain reaction–restriction fragment–length polymorphism and by enzyme-linked immunosorbent assay. The full VP1 genomic region of poliovirus isolates was sequenced with an ABI Prism BigDye Terminator Cycle Sequencing Ready Reaction kit and an automated DNA sequencer. Sequence data were compared with those of reference strains (GenBank). As a member of the Global Polio Laboratory Network, the National Polio Laboratory has annually reported the laboratory test results of poliovirus to WHO.

### Serological surveys

After one month of three rounds of SIAs, a serological survey was conducted in Ngawa County. Those who visited Ngawa County Hospital for a blood extraction for reasons not related to the polio investigation were invited to participate. An additional 2-ml blood sample was collected from each subject by venipuncture. Neutralization antibodies were determined by a microneutralization assay with authentic Sabin strains in accordance with WHO guidelines. A serum sample was considered positive if the neutralization antibody level was present at a dilution ≥1∶8.

### Ethical considerations

This study was approved by the China CDC institutional review board (IRB). The guardians of the individuals in this manuscript have given informed consent (as outlined in PLOS consent form) regarding publication of VDPV infection with potentially identifiable information, after study staffs explain fully to guardian about the purpose of the study, and the risks and benefits of publication. This kind of informed consent was securely filed in the individual's case notes.

## Results

### Vaccine-derived polioviruses cases

Information on outbreak case patients and their contacts is shown in table 1. The index patient (Case 1) was a 9-month-old boy born on November 23, 2010. On August 17, the patient developed fever and cough; on August 20, he developed bilateral weakness of lower extremities. The patient was hospitalized one month later (September 19), and the case was reported as having AFP on September 20. Epidemiologic investigations were conducted, and two inadequate stool specimens were obtained from the patient on September 20 and September 22. Type Π Sabin-related poliovirus strains were isolated from two specimens with 5 nt VP1 substitutions.

**Table 1:** Descriptive characteristics of case patients and close contacts in an outbreak of type Π cVDPVs in Ngawa County, Ngawa Tibetan and Qiang Autonomous Prefecture, Sichuan Province, China, August 2011–February 2012.

Group, No	County	Sex	Age (Years)	Doses of OPV received	Date of paralysis onset	Date of specimen collection	Poliovirus type	Final diagnosis
Case 1	Ngawa	Male	0.8	0	Aug 20, 2011	20–22 Sep, 2011	Π	clinical compatible polio case
Case 2	Ngawa	Female	0.9	0	Oct 15, 2011	18–19 Oct, 2011	Π	VDPV case
Case 3	Ngawa	Female	1	0	Jan 7, 2012	19–21 Jan, 2012	Π	VDPV case
Case 4	Ngawa	Male	0.8	0	Feb 6, 2012	23–24 Feb, 2012	Π	VDPV case

The second case patient (Case 2) was a 10-month-old girl with onset of fever on 14 Oct and of bilateral leg paralysis on Oct 15. On Oct 17, she was taken to Ngawa Prefecture People's Hospital where she was reported as having AFP. Type Π VDPV (6 nt VP1 substitutions) strains were isolated from stool specimens collected on Oct 18 and Oct 19. The third case patient (Case 3) was a 12-month-old girl. She developed fever and diarrhea on Dec 24, 2011 and right leg paralysis on Jan 7, 2012, and she was seen at township central clinic and Ngawa Prefecture People's Hospital. On Jan 16, because she had shown little improvement, she was taken to Huaxi second affiliated hospital of Sichuan university and was reported as having AFP. Type Π VDPV (8 nt VP1 substitutions) strains were isolated from stool specimens collected on Jan 19 and Jan 21. The fourth case patient (Case 4) was a 9-month-old boy with bilateral leg paralysis on Feb 6, and he was seen at Ngawa Prefecture people's hospital on Feb 21. On Feb 22, because he had shown little improvement, he was taken to Chengdu Hospital of Infectious Disease and was reported as having AFP. Type Π VDPV (11–12 nt VP1 substitutions) strains were isolated from stool specimens collected on Feb 23 and Feb 24.

All 4 case patients lived in Ngawa County and had not been immunized. The index case was diagnosed as clinical compatible polio case (poliovirus strain with 5 nt VP1 substitutions), and the other case patient were diagnosed as VDPV cases by provincial PEC.

### Contacts of case patients

During Feb 7–10, stool specimens were collected from 150 close contacts of case patients. A type Π VDPV strain was isolated from one other child (Case 3-c1) who lived in a neighboring village of the third case patient. A type Ш poliovirus strain (8 nt VP1 substitutions) was also isolated from one close contact of the third case patient.

### Genetic characterization of Vaccine-derived polioviruses

The first virus isolated from index patient was a type Π poliovirus strain, differing from the parental Sabin Π OPV strain with 5 nt VP1 nucleotides. VP1 sequences of isolates from case patients and isolate from close contacts in the community demonstrated that all were closely related poliovirus/VDPVs. Genetic sequencing showed that the isolates diverged from the VP1 region of the type Π Sabin strain by 5–12 nt nucleotides and shared the same 5 nt VP1 substitutions. Based on genetic sequencing and their VP1 relationship, they can be considered to be one single lineage of cVDPVs.

### Non-polio acute flaccid paralysis cases

**Figure 1:**
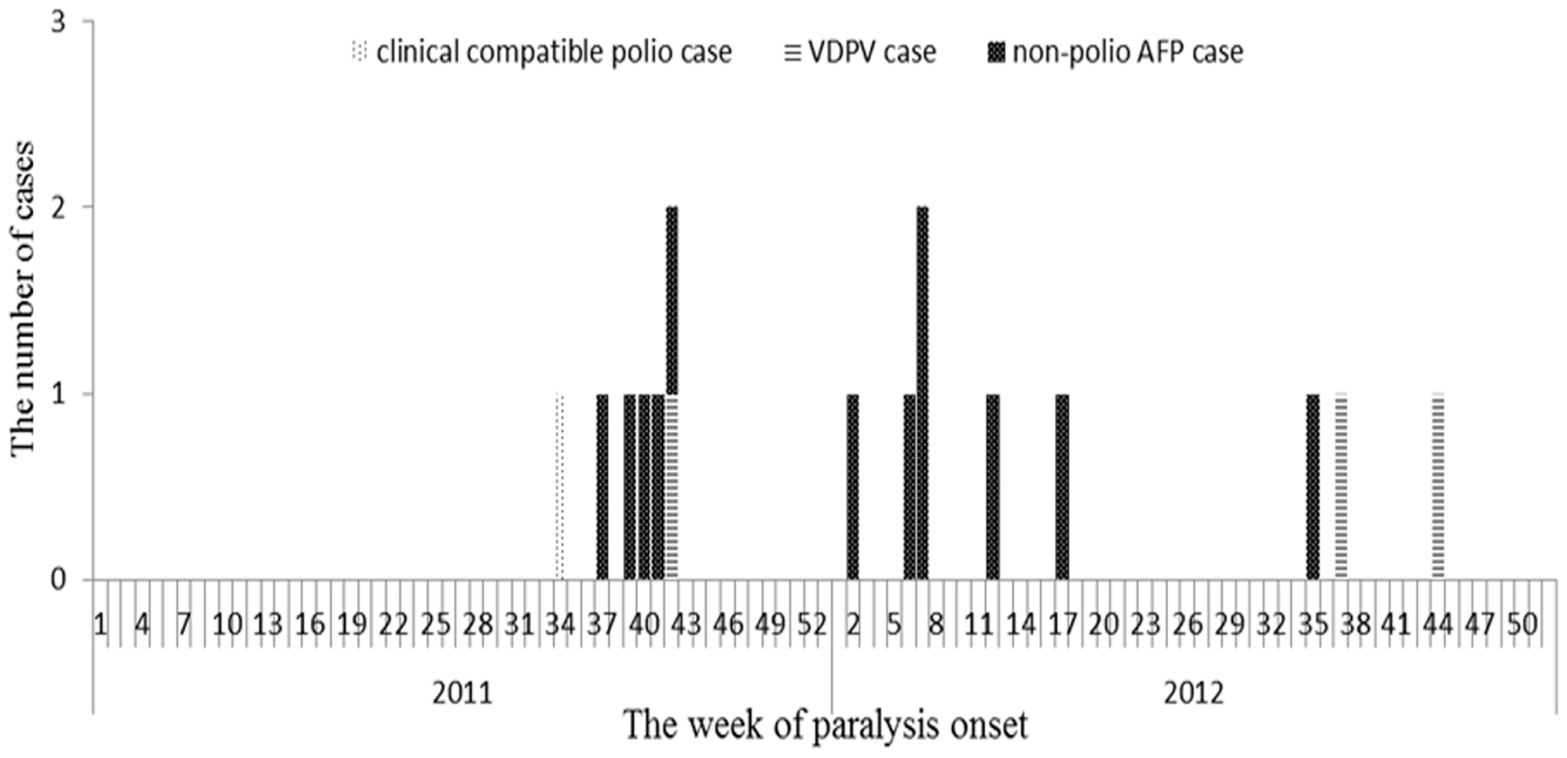
Weekly distribution of AFP cases <15 years of age by classification in 2011 and 2012 in Ngawa Tibetan and Qiang Autonomous Prefecture, Sichuan Province, China. Note: VDPV, vaccine-derived poliovirus; AFP, acute flaccid paralysis.

Totally, 7 AFP cases were reported in Ngawa Tibetan and Qiang Autonomous Prefecture in 2011, including one clinical compatible polio case, one VDPV case and 5 non-polio AFP cases all of whom were paralyzed after the index case (Fig. 1). Of the 5 non-polio AFP cases, 2 (40%) were diagnosed as non-polio enteroviruses (NPEV) infection, 1 (20%) with Guillain Barré syndrome, 1 (20%) with hypokalemic periodic paralysis and 1 (20%) with transverse myelitis.

A total of 9 AFP cases were reported in 2012, including 2 VDPV cases and 7 non-polio AFP cases all of whom were paralyzed after the last VDPV case (Fig. 1). All the 7 non-polio AFP cases were diagnosed as transverse myelitis.

### Risk assessment

During 6–10 February, 2012, a total of 210 children younger than 5 years of age were selected randomly in Ngawa County immediately after the outbreak was confirmed, for the purpose of evaluating OPV coverage. Three doses of OPV coverage was estimated to be 91% by routine immunization, and the measles vaccine coverage was 75%.

Of the 7 AFP cases (all >6 months) reported in Ngawa Tibetan and Qiang Autonomous Prefecture in 2011, all the 2 polio cases and 40% (2/5) non-polio AFP cases did not receive OPV immunization. Of the 9 AFP cases reported in 2012, all the 2 polio cases and 14.3% (1/7) non-polio AFP cases did not receive OPV immunization.

### Supplementary immunization activities

**Figure 2:**
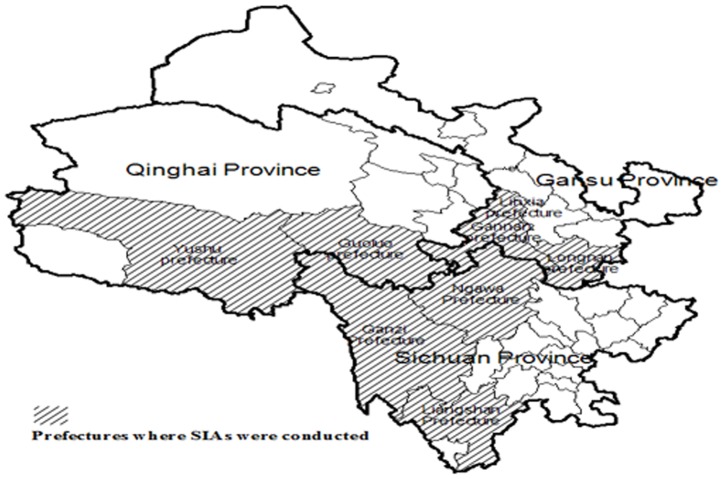
Distribution of prefectures where SIAs were conducted in Sichuan, Qinghai and Gansu provinces. Note: SIAs, supplementary immunization activities.

SIAs were conducted in Ngawa Tibetan and Qiang Autonomous Prefecture and neighboring prefectures, including Ganzi and Liangshan prefectures in Sichuan Province, Guoluo and Yushu prefectures in Qinghai Province, as well as Linxia, Gannan and Longnan prefectures in Gansu Province (Table 2 and Fig. 2).

**Table 2:** Rounds, dates, target population, and numbers of persons immunized for SIAs by province, 2012.

Province	Prefecture/city	Target population	Vaccination round	Date	No. immunized
Sichuan	Ngawa, Ganzi and Liangshan	2 mths–14 yrs	1	Feb 27–Mar 2	1,465,515
Sichuan	Ngawa, Ganzi and Liangshan	2 mths–14 yrs	2	Mar 31–Apr 4	1,495,877
Sichuan	Ngawa, Ganzi and Liangshan	2 mths–14 yrs	3	May 2–6	1,437,176
Qinghai	Guoluo and Yushu prefcture	2 mths–5 yrs	1	Feb 27–Mar 2	56,759
Qinghai	Guoluo and Yushu prefcture	2 mths–5 yrs	2	Mar 31–Apr 4	60,195
Gansu	Linxia and Gannan prefecture	2 mths–14 yrs	1	Feb 27–Mar 3	545,192
Gansu	Longnan prefecture	2 mths–6 yrs	1	Feb 27–Mar 3	108,698
Gansu	Linxia and Gannan prefecture	2 mths–14 yrs	2	Mar 27–Apr 1	553,170
Gansu	Longnan prefecture	2 mths–6 yrs	2	Mar 27–Apr 1	109,474

With the leadership of Sichuan government, multi-sectoral coordination mechanism was launched to organize SIAs. The staffs at all levels were trained to assure the quality of SIAs, including planning SIA, overseeing obligation and responsibility of staffs, providing technical guidelines, and monitoring adverse events of OPV. Community leaders went from house to house to register the target children for SIAs and to provide information about the vaccination campaign. Before the vaccination campaigns began, information about the vaccination campaign was disseminated through television, radio, and newspapers. Local political and religious leaders, community elders, and volunteers also helped disseminating the public health message.

Three rounds of SIAs were conducted in Sichuan Province targeting children younger than 15 years of age from February to May 2012, with a total of 4.4 million doses of OPV administered. Two rounds of SIAs were conducted in other two provinces.

### Interruption of transmission

Since March 2012, stool specimens from children with AFP throughout Sichuan Province have been negative for the outbreak strain, including specimens from 9 children with AFP who had onset of paralysis during March–December 2012. The reported incidence rate of non-polio AFP case in Ngawa Tibetan and Qiang Autonomous Prefecture was 5.04/100,000 among children younger than 15 years of age, and the collection rate of adequate stool specimens (88.9%) met WHO requirements. Comparatively, the reported incidence rate of non-polio AFP cases is 3.89/100,000 among children younger than 15 years of age, and the rate of adequate stool specimens was 85.7% (6/7) in 2011.

Externally surveyed coverage rates were assessed in three randomly selected townships, three randomly selected schools and one randomly selected market. Convenience samples were obtained from 210 children in these places. Coverage rates for each round of SIAs exceeded 98% in Ngawa County. After three rounds of SIAs, 153 blood specimens were collected from healthy children in Ngawa County in June 2012. The rate of a positive test for neutralization antibody against type Ι, Π and Ш poliovirus were 98.7%, 99.4% and 99.4%, respectively (Table 3).

**Table 3:** Antibody seropositivity of type I, type Π, and type 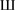 by age group in Ngawa County, Ngawa Tibetan and Qiang Autonomous Prefecture, Sichuan Province, China, 2012.

Age group(Yrs)	Total population (No.)	Type I	Type Π	Type Ш
		Seropositive N (%)	Seropositive N (%)	Seropositive N (%)
0–2	2	1 (50.0)	1 (50.0)	1 (50.0)
3–5	11	11(100.0)	11(100.0)	11(100.0)
6–8	72	72(100.0)	72(100.0)	72(100.0)
9–11	35	34(97.1)	35 (100.0)	35 (100.0)
12–14	33	33(100.0)	33(100.0)	33(100.0)
Total	153	151(98.7)	152 (99.4)	152 (99.4)

## Discussion

This report documents an outbreak of cVDPVs in China, which was certified as a poliomyelitis-free region in 2000. To our knowledge, this is the second outbreak of cVDPVs following the cVDPV outbreak in Guizhou Province in 2004 [7]. The outbreak occurred in a remote and poor rural area in southwest China. Evidence of the cVDPVs was limited to Ngawa Tibetan and Qiang Autonomous Prefecture, northwest of Sichuan Province. No other cases were found despite an intensive search, and circulation appeared to be limited to the areas with very low immunization coverage and population immunity. The outbreak was interrupted following three rounds of SIAs: the surveyed coverage rates for all three rounds of SIAs were greater than 98%; AFP surveillance system was enhanced with the incidence rate of non-polio AFP exceeding 2/100,000 among children younger than 15 years of age in 2012; and positivity rates for all three types of poliovirus were higher than 95% following SIAs.

Low immunization immunity due to poor OPV coverage in routine immunization among subpopulations was the major risk factor related with cVDPVs outbreak, as suggested by other studies [18]–[20]. Coverage with three doses of OPV was estimated to be 91%, and the reported routine immunization coverage was>95% in Ngawa Tibetan and Qiang Autonomous Prefecture. However, only 42.9% AFP cases and 60% non-polio AFP cases in 2011 had received ≥3 doses of OPV. The discrepancy of OPV coverage may be due to reluctance of provincial health departments to report low coverage because of performance assessment. It is difficult to determine the targeted children for immunization based on administrative coverage data because actual coverage is thought to be considerably lower than reported coverage [7], [21]. AFP surveillance can help identifying underserved communities for prioritization of routine immunization and SIA efforts. Surveys for coverage should be conducted routinely in high risk areas. In addition, all the polio cases identified in the outbreak had not received OPV. Absence of WPV transmission for more than a decade and inadequate services of routine immunization contributed to the accumulation of susceptible children, especially in remote poor areas of Ngawa County. Risk assessment and the surveillance of serologic antibodies against poliovirus should be conducted regularly to pinpoint high risk areas or subpopulations [11].

The outbreak investigation highlights the risk of cVDPVs in countries with high national immunization coverage but low coverage in subpopulations and immunity gaps in high-risk areas. Reasons for not vaccinating children vary among risk populations and include lack of knowledge and difficulty reaching by routine immunization among Madura rural population in Indonesia [18], religious beliefs for objecting immunization in Amish communities in the United States [20], and marginalization of Roma communities in Romania [19]. One of the major barriers to reaching children in Ngawa Tibetan and Qiang Autonomous Prefecture with polio vaccine and other health services is logistics. These communities reside in sparsely populated parts with no road access. In addition, the nomadic herders follow a seasonal pattern of movement with their cattle, with many of the settlements being temporary. Moreover, children residing in remote nomadic and scattered settlements lack access to routine immunization services. The last SIAs before paralysis onset of the index case were conducted in Ngawa Tibetan and Qiang Autonomous Prefecture in December 2009. The poor quality of routine immunization in these remote rural areas and lacking SIAs for more than 1 year may contribute to the accumulation of susceptible children. Therefore, it is speculated that immunity gap occurred recently, which is supported by the age distribution of polio cases in the outbreak that all polio cases were less than 1 year old. These subpopulations are also at high risk for WPV transmission following importation, as demonstrated by WPV outbreaks in the Xinjiang Uygur Autonomous Region, China and in Amish communities [11], [22]. Therefore, high coverage of routine immunization should be maintained in children until WPV transmission is globally eradicated, in addition, routine vaccination should be reinforced by preventive SIAs in high risk WPV importation areas to avoid the accumulation of susceptible individuals.

OPV is the preferred vaccine for polio eradication because it is easy to administer, spread to unvaccinated persons and is relatively inexpensive. However, in light of the risk of poliomyelitis from VDPV strains and VAPP, the continued use of OPV would compromise the goal of global eradication. A comprehensive strategic plan was developed to interrupt WPV transmission by the end of 2014 and to certify the world as polio free by 2018 [23]. One of the key actions was to replace current trivalent OPV with bivalent OPV containing only type Ι and Ш virus. The risk of cVDPVs emergence appears to be highest for the type Π OPV strain [12]–[14], even though type Π WPV has probably been eradicated [2]. To maintain population immunity to the type Π virus, the administration of at least one dose of inactivated poliovirus is recommended in routine immunization. Although the risk of cVDPVs outbreak after removing type Π from OPV is estimated to be low, the actual risk is difficult to predict, as the use of monovalent type Ι and Ш OPV in SIAs had widened the immunity gaps against type Π poliovirus in Nigeria [24]. The probability of cVDPVs outbreak in China would be much greater than that of most other countries, merely because of its large population and immunity gaps in subpopulations. Immunogenicity against type Π virus should be evaluated for the sequential procedure of IPV and bivalent OPV (Ι+Ш). The development of future polio immunization policies will require careful consideration of both the risks and costs of VDPVs infection based on research results of immunogenicity and vaccine costs.

It appears that the Sabin type Π OPV strain is easier to spread among unimmunized populations and has higher virulence, as shown by the more frequent reports of type Π poliovirus [12], [14], particularly in areas with high densities of unimmunized population and poor sanitation. Therefore, type Π VDPV is defined as Sabin-related poliovirus with ≥6 nt VP1substitutions, whereas type Ι and Ш VDPV with ≥10 nt VP1 substitutions. The demarcation of 6 or 10 VP1 divergence does not imply that isolates with less divergence would lack the capacity for person-to-person transmission, as it is likely that the critical attenuating mutations keep well before nucleotide substitutions reach the demarcation level [19], [25]. In our study, the virus isolated from the index case only had 5 nt VP1substitutions from the Sabin strain and the case did not meet the definition for a VDPV case. Therefore, an epidemiological survey for polio compatible case should be conducted if vaccine related poliovirus is detected, although isolated virus does not meet the VDPV definition. Through multi-sectoral cooperation under the leadership of provincial government, the timely implementation of three rounds of SIAs stopped the outbreak of cVDPVs, however, the outbreak demonstrates the necessity for polio-free countries to maintain high population immunity in high risk areas, to prevent the transmission of both cVDPVs and imported WPV. Prompt investigation and emergency response are recommended similarly to that proposed for WPV importation [4]. Risk assessment should be conducted regularly to pinpoint the high risk areas or subpopulations, with SIAs developed if necessary.
